# Reducing Initial Loss to Follow-up Among People With Bacteriologically Confirmed Tuberculosis: LINKEDin, a Quasi-experimental Study in South Africa

**DOI:** 10.1093/ofid/ofad648

**Published:** 2023-12-18

**Authors:** Sue-Ann Meehan, Anneke C Hesseling, Andrew Boulle, Jolene Chetty, Lucy Connell, Nomthandazo J Dlamini-Miti, Rory Dunbar, Karen Du Preez, Gavin George, Graeme Hoddinott, Karen Jennings, Florian M Marx, Vanessa Mudaly, Pren Naidoo, Neo Ndlovu, Jacqueline Ngozo, Mariette Smith, Michael Strauss, Gaurang Tanna, Nosivuyile Vanqa, Arne von Delft, Muhammad Osman

**Affiliations:** Desmond Tutu TB Centre, Department of Paediatrics and Child Health, Faculty of Medicine and Health Sciences, Stellenbosch University, Cape Town, South Africa; Desmond Tutu TB Centre, Department of Paediatrics and Child Health, Faculty of Medicine and Health Sciences, Stellenbosch University, Cape Town, South Africa; Centre for Infectious Disease Epidemiology and Research, School of Public Health and Family Medicine, Faculty of Health Sciences, University of Cape Town, Cape Town, South Africa; Department of Health and Wellness, Health Intelligence Directorate, Western Cape Government, Cape Town, South Africa; Interactive Research and Development South Africa (IRD SA), Sandton, Johannesburg; Right to Care South Africa, Helen Joseph Hospital, Johannesburg, South Africa; Right to Care South Africa, Helen Joseph Hospital, Johannesburg, South Africa; Desmond Tutu TB Centre, Department of Paediatrics and Child Health, Faculty of Medicine and Health Sciences, Stellenbosch University, Cape Town, South Africa; Desmond Tutu TB Centre, Department of Paediatrics and Child Health, Faculty of Medicine and Health Sciences, Stellenbosch University, Cape Town, South Africa; Health Economics and HIV and AIDS Research Division (HEARD), University of KwaZulu-Natal, Durban, South Africa; Division of Social Medicine and Global Health, Lund University, Lund, Sweden; Desmond Tutu TB Centre, Department of Paediatrics and Child Health, Faculty of Medicine and Health Sciences, Stellenbosch University, Cape Town, South Africa; City of Cape Town Health Department, Cape Town, South Africa; Desmond Tutu TB Centre, Department of Paediatrics and Child Health, Faculty of Medicine and Health Sciences, Stellenbosch University, Cape Town, South Africa; Division of Tropical Medicine, Center for Infectious Diseases, Heidelberg University Hospital, Heidelberg, Germany; DSI-NRF South African Centre of Excellence in Epidemiological Modelling and Analysis (SACEMA), Stellenbosch University, Stellenbosch, South Africa; Department of Health and Wellness, Western Cape Government, Cape Town, South Africa; Bill and Melinda gates Foundation, Johannesburg, South Africa; Right to Care South Africa, Helen Joseph Hospital, Johannesburg, South Africa; Kwa-Zulu Natal Department of Health and Wellness, Pietermaritzburg, South Africa; Centre for Infectious Disease Epidemiology and Research, School of Public Health and Family Medicine, Faculty of Health Sciences, University of Cape Town, Cape Town, South Africa; Department of Health and Wellness, Health Intelligence Directorate, Western Cape Government, Cape Town, South Africa; Health Economics and HIV and AIDS Research Division (HEARD), University of KwaZulu-Natal, Durban, South Africa; Bill and Melinda gates Foundation, Johannesburg, South Africa; Desmond Tutu TB Centre, Department of Paediatrics and Child Health, Faculty of Medicine and Health Sciences, Stellenbosch University, Cape Town, South Africa; Centre for Infectious Disease Epidemiology and Research, School of Public Health and Family Medicine, Faculty of Health Sciences, University of Cape Town, Cape Town, South Africa; Department of Health and Wellness, Health Intelligence Directorate, Western Cape Government, Cape Town, South Africa; Desmond Tutu TB Centre, Department of Paediatrics and Child Health, Faculty of Medicine and Health Sciences, Stellenbosch University, Cape Town, South Africa; School of Human Sciences, University of Greenwich, London, UK

**Keywords:** initial loss to follow-up, tuberculosis

## Abstract

Every person diagnosed with tuberculosis (TB) needs to initiate treatment. The World Health Organization estimated that 61% of people who developed TB in 2021 were included in a TB treatment registration system. Initial loss to follow-up (ILTFU) is the loss of persons to care between diagnosis and treatment initiation/registration. LINKEDin, a quasi-experimental study, evaluated the effect of 2 interventions (hospital recording and an alert-and-response patient management intervention) in 6 subdistricts across 3 high–TB burden provinces of South Africa. Using integrated electronic reports, we identified all persons diagnosed with TB (Xpert MTB/RIF positive) in the hospital and at primary health care facilities. We prospectively determined linkage to care at 30 days after TB diagnosis. We calculated the risk of ILTFU during the baseline and intervention periods and the relative risk reduction in ILTFU between these periods. We found a relative reduction in ILTFU of 42.4% (95% CI, 28.5%–53.7%) in KwaZulu Natal (KZN) and 22.3% (95% CI, 13.3%–30.4%) in the Western Cape (WC), with no significant change in Gauteng. In KZN and the WC, the relative reduction in ILTFU appeared greater in subdistricts where the alert-and-response patient management intervention was implemented (KZN: 49.3%; 95% CI, 32.4%–62%; vs 32.2%; 95% CI, 5.4%–51.4%; and WC: 34.2%; 95% CI, 20.9%–45.3%; vs 13.4%; 95% CI, 0.7%–24.4%). We reported a notable reduction in ILTFU in 2 provinces using existing routine health service data and applying a simple intervention to trace and recall those not linked to care. TB programs need to consider ILTFU a priority and develop interventions specific to their context to ensure improved linkage to care.

Tuberculosis (TB) is the leading cause of death from a single infectious disease [1]. In the END TB strategy, all member states of the World Health Organization (WHO) committed to a world free of TB, to be achieved through reductions in TB incidence, mortality, and the catastrophic costs faced by TB-affected households [2]. A pillar of this strategy included integrated, patient-centred care and prevention, with an emphasis on early diagnosis and treatment of all people with TB [2].

After accessing TB tests, every person with TB (PWTB) needs to receive their results, initiate TB treatment, and be recorded in a TB reporting system to enable accurate surveillance and monitoring and evaluation of TB care. Initial loss to follow-up (ILTFU) has been defined as the loss of persons to care following their diagnosis of TB, before their inclusion in a TB reporting system. People who are ILTFU are at elevated risk of morbidity and mortality [3, 4], and untreated disease contributes to ongoing transmission of *Mycobacterium tuberculosis* [3, 5]. In 2021, 39% (4.2 million people) of those who developed TB were not treated and/or not recorded in a TB registration system [1]. ILTFU is estimated to be between 4% and 38% globally, 18% in Africa [6] and 17.1% in South Africa [7].

South Africa is a high–TB burden country, with an estimated incidence of 304 000 TB cases in 2021, with >120 000 either not diagnosed or not included in routine reporting [1]. In South Africa, 12% of persons with drug-susceptible TB [7] and 37% of persons with drug-resistant TB [8] are lost between diagnosis and TB registration. Reducing ILTFU in South Africa is a priority to improve TB control. Interventions addressing ILTFU could have a substantial impact on the TB epidemic. These persons have accessed health care services and have a laboratory-confirmed diagnosis of TB, yet have not been linked to a TB treatment facility for registration and initiation of treatment. Few studies have evaluated interventions addressing ILTFU across diverse settings. The LINKEDin study evaluated the effect of 2 interventions to reduce ILTFU at the hospital and primary health care facility levels in 3 high-burden provinces in South Africa.

## METHODS

### Study Design

We conducted a quasi-experimental study to investigate the effect of (1) a hospital-based recording intervention that linked PWTB to standard hospital management and referral processes and (2) an alert-and-response patient management intervention to reduce ILTFU among individuals routinely diagnosed with TB. We defined ILTFU as all persons diagnosed with TB (Xpert MTB/RIF positive) for whom there was no evidence of linkage to a public TB treatment facility for TB registration and treatment within 30 days of the date of diagnosis.

To measure the effect of these interventions, we calculated the relative reduction in ILTFU between the 3-month baseline period (October 2018 to December 2018) and the intervention period (January 2019 to December 2020). Prospective data were collected for both periods.

Using integrated electronic reports, we identified all persons routinely diagnosed by Xpert MTB/RIF, as per standard of care in South Africa, in the hospital and at primary health care (PHC) facilities and prospectively determined ILTFU.

### Study Setting

The study was implemented in KwaZulu-Natal (KZN), Gauteng (GP), and the Western Cape (WC) provinces, 3 of the highest TB-burdened provinces in South Africa [9]. Study site selection and implementation were in consultation with provincial and district TB program managers. We identified a district within each province: Ugu in KZN, City of Johannesburg in GP, and City of Cape Town in the WC (Supplementary Table 1, key differences in setting). Two subdistricts within each district were then selected. We liaised with local TB program managers, who used their routine TB data—TB burden and estimated ILTFU among PWTB—to help guide selection of facilities for inclusion. Willingness of subdistrict and facility mangers to be included in the study was also considered.

In South Africa, TB investigation, diagnosis, and treatment initiation take place at any level of care in the public health care system, but TB reporting systems are maintained at designated TB treatment sites. This includes PHC facilities, where persons with TB receive treatment on an outpatient basis, and specialized TB hospitals, where persons who require hospitalization for TB are treated. PWTB initiated on TB treatment in general hospitals needed to be linked to a PHC facility for recording and continuation of their TB treatment.

Unique to the WC, the Department of Health houses a provincial health data centre (PHDC) that harmonizes all electronic patient health data from all public sector services in the province, producing a single patient record [10]. The PHDC generates disease-specific reports and, for TB, collates data from laboratory sources (including smear, culture, or Xpert MTB/RIF), pharmacy or clinical records, TB treatment registers, and TB-specific elements recorded in electronic data systems at the PHC or hospital level [10].

### Interventions

Within each district, we implemented a hospital-recording intervention in 1 subdistrict and an alert-and-response patient management intervention in the second subdistrict (Table 1).

**Table 1.** Health Facilities per Intervention Type by District and Subdistrict Included in the LINKEDin Study

District	Subdistrict Name	Intervention Type	Hospital	PHC TB Treatment Facilities
Ugu (KZN)	Umdoni	Hospital recording	GJ Crookes (district hospital, ∼300 beds)	N/A
	Ray Nkonyeni	Alert-and-response patient management	Gamalakhe^a^ (CHC)	10 surrounding PHC facilities
City of Johannesburg (GP)	Region D	Hospital recording	Chris Hani Baragwanath (tertiary hospital, ∼3200 beds)	N/A
	Region E	Alert-and-response patient management	Edenvale (district hospital, ∼230 beds)	9 surrounding PHC facilities
City of Cape Town (WC)	Tygerberg	Hospital recording	Tygerberg (tertiary hospital ∼1899 beds)	N/A
	Khayelitsha	Alert-and-response patient management	Khayelitsha (district hospital, ∼230 beds)	13 surrounding PHC facilities

Abbreviations: CHC, community health center; KZN, KwaZulu Natal; PHC, primary health care; TB, tuberculosis; WC, Western Cape.

^a^Gamalakhe is a large CHC but used as a proxy for a hospital in this study at the request of the KZN Department of Health as 10 surrounding PHC facilities refer to it.

#### Hospital-Recording Intervention

Study-appointed data clerks were placed at each hospital and used the routine data system available in the province. In KZN and GP, they used “Xpert Alerts” (a weekly National Health Laboratory Service [NHLS] line list of all people newly diagnosed using the Xpert MTB/RIF ultra-assay). These are sent from NHLS to health district offices for further distribution to health facilities to improve patient management. In the WC, the clerk used the PHDC [10] to identify all newly diagnosed PWTB.

Lists of newly diagnosed PWTB were shared with hospital staff to confirm whether patients were initiated on treatment in the hospital. There were no additional interventions to assist patients to link to a TB treatment facility once discharged from the hospital, beyond the routine referral mechanisms already in place.

#### Alert-and-Response Patient Management Intervention

Clerks based at the hospitals in the Ray Nkonyeni, Region E, and Khayelitsha subdistricts used Xpert Alerts (KZN/GP) and PHDC (WC) to identify all persons routinely diagnosed with TB at the selected PHC facilities in addition to those identified at the hospital. They monitored linkage and registration at TB treatment facilities for all persons identified with TB. In KZN and GP, they used the electronic TB treatment register (TIER.Net) to check for a TB treatment start date. TIER.Net is an electronic register used to capture patient-level HIV and TB information at the facility level and is integrated with the district health information system (DHIS) for reporting various program data from subdistricts to the national level [11, 12]. In the WC, they used the PHDC to check for evidence of linkage to and registration at a TB treatment facility. All patients eligible to link but with no evidence of linkage were followed up by a short message service (SMS), followed by a phone call and then creating a referral for a community-based health worker (CHW) to do a home visit to facilitate linkage. Persons with TB who had no telephonic details were immediately referred to a CHW. In KZN and GP, SMS messaging and telephone calls were done by data clerks using mobile phones. In the WC, the capabilities within the PHDC enabled SMS messaging initially, and later telephone calls to be made directly via the PHDC.

### Data Collection

Post intervention, we used the electronic health records to determine ILTFU for the baseline and intervention periods. In KZN and GP, we used matching algorithms to compare individuals with a TB diagnosis against TIER.Net. Linkage to care was confirmed when the PWTB had a TB treatment start date recorded in TIER.Net. Individuals with no TB treatment initiation date or a date >30 days after their date of diagnosis in TIER.Net (TB register) were defined as ILTFU. To account for patient movement between facilities, we searched for PWTB in TIER.Net at the district level for the baseline and intervention periods. To validate the matching algorithm output, data clerks in KZN searched TIER.Net for TB treatment start dates for everyone labeled ILTFU. We were unable to follow this process in GP as permission to access data beyond the subdistrict was not granted beyond the intervention phase. In the WC, linkage to care was confirmed when the PWTB had evidence in the PHDC of accessing a TB treatment facility anywhere in the province for TB treatment within 30 days.

As LINKEDin was embedded within health services and should reflect the routine TB program, we included data from the period April to June 2020 (coronavirus disease 2019 [COVID-19] lockdown), when study field staff were withdrawn, but routine hospital and PHC activities continued, with restrictions (Supplementary Tables 3–5, analysis excluding the COVID-19 lockdown period).

### Statistical Analysis

We conducted a before-and-after analysis comparing ILTFU in the baseline and intervention phases of the study. We calculated the risk of ILTFU in both periods and conducted 1-sided *t* tests to assess if there was a reduction between the baseline and intervention periods. We calculated the relative risk reduction in ILTFU between the intervention and baseline periods equivalent to 1-relative risk. In the WC, through the PHDC, we had data on all PWTB (confirmed and clinical diagnoses) and conducted an additional analysis for the WC (Supplementary Table 2). SAS software, version 9.4 (SAS Institute Inc., Cary, NC, USA), was used for data analysis.

### Ethics

The study was approved by the Health Research Ethics Committee at Stellenbosch University (N18/07/069), the University of the Witwatersrand (M190128), and the relevant provincial departments of health. The authors have no conflict of interest to declare.

### Patient Consent

This study does not include factors necessitating patient consent.

## RESULTS

During the intervention period, there were 1999 PWTB diagnosed in KZN, 5399 in GP, and 9359 in the WC (Table 2) at the selected facilities. The proportion of PWTB diagnosed in hospitals was 37.8% in KZN, 29.2% in GP, and 20.7% in the WC, while the proportion of ILTFU diagnosed in the hospital was 42.1% in KZN, 56.8% in GP, and 46.7% in the WC.

**Table 2.** Relative Reduction in ILTFU Between Baseline and Intervention Periods per Province

	Oct–Dec 2018		Jan 2019–Dec 2020		Relative Reduction ILTFU (95% CI), %
	Newly Diagnosed PWTB, No.	ILTFU, No. (%) (95% CI, %)	Newly Diagnosed PWTB, No.	ILTFU, No. (%) (95% CI, %)	
KwaZulu-Natal	327	81 (24.8) (20.1–29.4)	1999	285 (14.3) (12.7–15.8)	42.4 (28.5–53.7)
Gauteng	921	292 (31.7) (28.7–34.7)	5399	1772 (32.8) (31.6–34.1)	−3.5 (−14.7 to 6.5)
Western Cape	1323	296 (22.4) (20.1–24.6)	9359	1627 (17.4) (16.6–18.2)	22.3 (13.3–30.4)

Abbreviations: ILTFU, initial loss to follow-up; PWTB, person with tuberculosis.

### Overall ILTFU Between Baseline and Intervention Periods Across Provinces

Following the interventions, we found a considerable relative reduction in ILTFU of 42.4% (95% CI, 28.5%–53.7%) in KZN and 22.3% (95% CI, 13.3%–30.4%) in WC. In GP, there was no change in ILTFU (Table 2). In the WC, an additional analysis not restricted to Xpert-confirmed TB showed a higher proportion of ILTFU but no difference in the relative reduction of ILTFU compared with the primary analysis (Supplementary Table 2).

### ILTFU Between the Baseline and Intervention Periods by Subdistricts Across Provinces

In KZN and WC, the relative reduction in ILTFU appeared greater in subdistricts where the alert-and-response patient management intervention was implemented compared with subdistricts where only the hospital-recording intervention was implemented. The relative reduction in KZN was 49.3% (95% CI, 32.4%–62.0%) vs 32.2% (95% CI, 5.4%–51.4%), and in the WC, it was 34.2% (95% CI, 20.9%–45.3%) vs 13.4% (95% CI, 0.7%–24.4%). In Gauteng, there was no relative reduction in ILTFU (Table 3).

**Table 3.** Relative Reduction in ILTFU Between Baseline and Intervention Periods by Subdistricts Across Provinces

		Oct–Dec 2018		Jan 2019–Dec 2020		Relative Reduction ILTFU (95% CI), %	*P* Value 1-Sided *T* Test^a^
		Newly Diagnosed Persons With TB, No.	ILTFU, No. (%) (95% CI, %)	Newly Diagnosed Persons With TB, No.	ILTFU, No. (%) (95% CI, %)		
KwaZulu Natal	Umdoni (hospital recording)	131	33 (25.2) (17.8–32.6)	790	135 (17.1) (14.5–19.7)	32.2 (5.4–51.4)	.0131
	Ray Nkonyeni (alert and response)	196	48 (24.5) (18.5–30.5)	1209	150 (12.4) (10.5–14.3)	49.3 (32.4–62)	<.0001
Gauteng	Region D (hospital recording)	713	208 (29.2) (25.8–32.5)	4099	1301 (31.7) (30.3–33.2)	−8.8 (−23.0 to 3.8)	.9170
	Region E (alert and response)	208	84 (40.4) (33.7–47.1)	1300	471 (36.2) (33.6–38.8)	10.3 (−7.4 to 25.1)	.1288
Western Cape	Tygerberg (hospital recording)	761	185 (24.3) (21.3–27.4)	5095	1073 (21.1) (19.9–22.2)	13.4 (.7–24.4)	.0251
	Khayelitsha (alert and response)	562	111 (19.8) (16.5–23.0)	4264	554 (13) (12.0–14.0)	34.2 (20.9–45.3)	<.0001

^a^One-sided *t* test: based on the null hypothesis that the percent ILTFU was not reduced from baseline to intervention.

Abbreviations: ILTFU, initial loss to follow-up; TB, tuberculosis.

### ILTFU in Subdistricts Where the Alert-and-Response Patient Management Intervention Was Implemented

In subdistricts where the alert-and-response intervention was implemented, there appeared to be greater relative reductions in ILTFU in the PHC facilities surrounding the hospital compared with in the hospital itself (KZN: 56.9%; 95% CI, 41.1%–68.5%; vs 3.4%; 95% CI, –103.7% to 54.2%; and WC: 52.4%; 95% CI, 40.9%–61.7%; reduction vs an increase of 11.6%; 95% CI, –61.4% to 22.9%) (Table 4).

**Table 4.** Relative Reduction in ILTFU Between Baseline and Intervention Periods by Place of Diagnosis for Subdistricts by Intervention Type

		Oct–Dec 2018		Jan 2019–Dec 2020		Relative Reduction ILTFU (95% CI), %	*P* Value 1-Sided *T* Test^a^
		Newly Diagnosed PWTB, No.	ILTFU, No. (%) (95% CI, %)	Newly Diagnosed PWTB, No.	ILTFU, No. (%) (95% CI, %)		
Subdistricts implementing the hospital-recording intervention (no intervention in surrounding facilities)							
Umdoni (KwaZulu-Natal)	GJ Crookes Hosp	65	23 (35.4) (23.8–47)	345	73 (21.2) (16.8–25.5)	40.2 (12–59.4)	.0141
	Surrounding PHC facilities	66	10 (15.2) (6.5–23.8)	445	62 (13.9) (10.7–17.2)	8 (−70.2 to 50.3)	.3989
Region D (Gauteng)	CH Baragwanath Hosp	169	94 (55.6) (48.1–63.1)	1167	746 (63.9) (61.2–66.7)	−14.9 (−32.4 to 0.2)	.9784
	Surrounding PHC facilities	544	114 (21.0) (17.5–24.4)	2932	555 (18.9) (17.5–20.3)	9.7 (−7.5 to 2.1)	.1420
Tygerberg (Western Cape)	Tygerberg Hospital	173	74 (42.8) (35.4–50.1)	1132	509 (45) (42.1–47.9)	−5.1 (−26.4 to 12.5)	.7053
	Surrounding PHC facilities	588	111 (18.9) (15.7–22)	3963	564 (14.2) (13.1–15.3)	24.6 (9.4–37.3)	.0015
Subdistricts implementing the alert-and-response patient management intervention							
Ray Nkonyeni (KwaZulu-Natal)	Gamalakhe CHC	59	7 (11.9) (3.6–20.1)	410	47 (11.5) (8.4–14.5)	3.4 (−103.7 to 54.2)	.4648
	Surrounding PHC facilities	137	41 (29.9) (22.3–37.6)	799	103 (12.9) (10.6–15.2)	56.9 (41.1–68.5)	<.0001
Region E (Gauteng)	Edenvale Hospital	59	43 (72.9) (61.5–84.2)	409	259 (63.3) (58.7–68)	13.1 (−3.2 to 26.9)	.0668
	Surrounding PHC facilities	149	41 (27.5) (20.3–34.7)	891	212 (23.8) (21.0–26.6)	13.5 (−15.1 to 35)	.1728
Khayelitsha (Western Cape)	Khayelitsha Hospital	79	22 (27.8) (18.0–37.7)	808	251 (31.1) (27.9–34.3)	−11.6 (−61.4 to 22.9)	.7261
	Surrounding PHC facilities	483	89 (18.4) (15.0–21.9)	3456	303 (8.8) (7.8–9.7)	52.4 (40.9–61.7)	<.0001

^a^One-sided *t* test: based on the null hypothesis that the percent ILTFU was not reduced from baseline to intervention.

Abbreviations: ILTFU, initial loss to follow-up; PHC, primary health care; PWTB, person with tuberculosis.

### ILTFU in Subdistricts Where Only the Hospital-Recording Intervention Was Implemented

GJ Crookes Hospital, KZN, had a 40.2% (95% CI, 12.0%–59.4%) relative reduction in ILTFU, while no change was seen in hospitals in GP and the WC. In the PHC facilities surrounding Tygerberg Hospital (WC), there was a relative reduction in ILTFU (24.6%; 95% CI, 9.4%–37.3%) (Table 4).

## DISCUSSION

LINKEDin was an operational research study aimed to reduce ILTFU among PWTB in South Africa. With limited data on reducing ILTFU, LINKEDin provides important findings across 3 heterogeneous contexts in South Africa.

We demonstrated successful reductions in ILTFU in KZN (from 24.8% to 14.3%) and the WC (from 22.4% to 17.4%). The study was implemented in rural subdistricts of KZN, where PHC facilities are further apart, and we hypothesize that PWTB may be more likely to access services within their communities, closest to their homes, where they are known. This may have made these persons easier to track. This, together with the much lower numbers of PWTB, compared with GP and the WC, may have made the manual process of tracking individuals easier and played a role in the reduction in ILTFU observed in KZN. In the WC, the PHDC enabled us to evaluate linkage beyond the district. This is especially important in South Africa, where there is frequent movement of people within and across provinces [13].

We did not show a reduction in ILTFU in GP overall (from 31.7% to 32.8%). This was potentially driven by the increase in ILTFU by 8.8% in Region D (the subdistrict where we implemented the hospital-recording intervention at the Chris Hani Baragwanath Academic Hospital [CHBAH], a large tertiary-level hospital). The numbers of PWTB in this subdistrict were much higher compared with those in Region E (subdistrict where the alert-and-response patient management intervention was implemented) and where we did find a relative reduction in ILTFU of 10.3%. The disparity across settings makes it extremely difficult to compare results across provinces. What is important to note is that irrespective of geographical location or access to automation, systematically identifying persons with TB and following them up using the data and systems available in each setting can reduce ILTFU.

There was a tendency toward a greater reduction in ILTFU in settings where the alert-and-response intervention was implemented compared with settings where the hospital-recording intervention was implemented. This implies that while there is some benefit to registering persons with TB in the hospital, additional patient-centered interventions to follow up with PWTB who fail to link to care soon after their diagnosis or discharge from hospital are vital. Previous studies that addressed patient referral and education [14] and combined patient education and telephonic follow-up [15] showed improved linkages from hospitals. For sustained impact, an emphasis on health system interventions that support existing services rather than activities that are externally supported are needed.

ILTFU was higher at hospitals (range, 21.2%–63.9%) compared with PHC facilities (range, 11.5%–23.8%). This is consistent with earlier work in South Africa, which showed that ILTFU was high (between 37% and 50%) among people diagnosed with TB in hospitals [16, 17]. Gamalakhe CHC (ILTFU was 11.5%) was used as a proxy for a hospital but is not comparable to other study hospitals, as the referral process to Gamalakhe CHC is more like a PHC facility referral process.

Reducing ILTU in hospitals is extremely challenging, and LINKEDin could not fully address this challenge, irrespective of the size or level of the hospital. ILTFU is specifically higher at tertiary-level hospitals where the number of people diagnosed with TB is considerably higher than at district-level hospitals. At CHBAH and Tygerberg hospitals, there were 1167 and 1132 people diagnosed with TB, respectively, during the intervention period, compared with 345 at GJ Crookes. ILTFU at CHBAH and Tygerberg was 63.9% and 45%, respectively, during the intervention period, compared with 21.2% at GJ Crookes. Previous studies have observed a similar phenomenon, whereby ILTFU is more likely at high-volume facilities [18] and in high-burden settings [19]. Previous data from Chris Hani Baragwaneth in 2001 demonstrated that only half of the TB patients referred to PHC facilities attended services within 2 weeks [16] and, following an intervention between 2003 and 2005, that >90% attended the PHC facility with the help of research staff [14]. Our findings differed as we only implemented the hospital-recording intervention at some hospitals and encountered additional complexities within the alert-and-response intervention. Studies in hospitals have shown high workload, staff shortages, and inadequate skills, resulting in insufficient information and health education for persons with TB and their caregivers [20], as well as a fragmented hospital information system without linkages [21], resulting in less-than-optimal linkage to care.

People diagnosed in the hospital are often sicker, diagnosed late, and therefore more likely to die before they link to a PHC facility [6]. They may also not have accessed a PHC facility previously and be unfamiliar with access to community-level care, thereby delaying linkage. Interventions to promote earlier diagnosis in primary health care are needed. An additional exacerbating factor is that a proportion of PWTB are discharged before their positive TB test result is known, with no systematic process at the hospital level for recall. Improved communication from hospital staff with an emphasis on navigating the organizational barriers in the health system is required to support better linkage for these patients [22, 23]. Future work that differentiates the point of diagnosis within the hospital (outpatient vs in-patient) and offers tailored engagement, as reported in a recent cohort from China [24], to PWTB and/or their caregivers during or before discharge is key. The South African Department of Health has launched the National Health Hotline (The National health hotline was implemented after preliminary data from the LINKEDin study showed promise that systematic follow up of TB patients could reduce ILTFU. The hotline is an independent intervention, implemented by NDoH, but not part of the LINKEDin study), which aims to improve contact with persons who test positive, trace, or unsuccessful for TB Xpert through communication of test results and improving linkage to care for access to treatment at a health facility. Having correct patient contact details is vital for the success of any intervention that promotes linkage [25], irrespective of setting, level of care, or patient volume.

### Practical Recommendations

The challenge of ILTFU can be addressed using setting-specific programmatic data to systematically identify and follow persons diagnosed with TB. This should be done using existing personnel and be embedded within the existing health system interventions. It is important that interventions to reduce ILTFU be part of the routine monitoring and evaluation of TB programs [26]. We recommend updating patient contact details at every health visit to ensure that patients who require additional support with linkage can be easily traced [22].

Interventions to address ILTFU should be prioritized for hospital-diagnosed patients. We recommend person-centered communication between the health care provider and the patient before discharge that includes practical advice on where and how to access a PHC facility for treatment and offers the PWTB an opportunity to ask questions and better understand their disease [27].

A major strength of our study was the implementation of interventions in diverse geographical and health care settings, embedded within the routine TB program. The use of existing resources within this operational research study demonstrates the feasibility of implementing the interventions. Despite varying reductions in ILTFU, we reported a notable reduction in ILTFU in 2 settings between the baseline and intervention periods.

A before-and-after study is vulnerable to temporal and other changes beyond the intervention, and we cannot attribute the successes solely to our interventions. The variation in sample sizes is a limitation for comparability across the settings. This, combined with the small changes in ILTFU in some settings, resulted in significant statistical uncertainty around some of the relative reduction estimates.

A limitation in our definition was that persons with TB who linked 30 days after diagnosis were categorized as ILTFU, irrespective of where they were diagnosed. This may have overestimated ILTFU. Further analysis is planned to address the time to linkage.

In Gauteng, we could not search for patients reported as ILTFU in the baseline or intervention periods in other subdistricts, as had been done in the other provinces. This has likely resulted in an overestimation of ILTFU and an underestimation of relative reductions in ILTFU in Gauteng.

We experienced severe limitations during the COVID-19 pandemic. For a quarter of 2020, no study staff were in place, and all study activities were suspended. Routine clinic activities continued, with many resources redirected away from TB toward COVID-19 services. We conducted an analysis excluding the period when there were no study staff in the field and saw no significant difference in the primary analysis (Supplementary Tables 3–5). Finally, we could not determine the wider impact of these interventions toward reducing community transmission; this presents an opportunity for further research, for example, modeling.

LINKEDin was embedded within routine health services and aimed to reduce ILTFU in 3 diverse settings in South Africa. The findings provide important lessons in each setting. By identifying all persons newly diagnosed with TB using existing routine health service data and applying a consistent intervention to trace and recall those not linked to care following diagnosis, we demonstrated an overall reduction in ILTFU of 49% in KZN and 34% in the WC. TB programs must consider ILTFU a priority and develop interventions specific to their settings. The use of operational research to test ILTFU interventions would address the contextual complexity in different settings. Unless there is a shift to include all persons diagnosed with TB in the routine reporting of TB, the TB treatment cohort will continue to exclude ILTFU.

## Supplementary Material

ofad648_Supplementary_Data

## Supplementary Data

Supplementary materials are available at *Open Forum Infectious Diseases* online. Consisting of data provided by the authors to benefit the reader, the posted materials are not copyedited and are the sole responsibility of the authors, so questions or comments should be addressed to the corresponding author.
