# Supplementary material for: Reducing Initial Loss to Follow-up Among People With Bacteriologically Confirmed Tuberculosis: LINKEDin, a Quasi-experimental Study in South Africa
Source: Open Forum Infect Dis. 2023 Dec 18;11(1):ofad648. doi: 10.1093/ofid/ofad648 (PMC10787480; doi:10.1093/ofid/ofad648)
Supplement: ofad648_Supplementary_Data [file ofad648_supplementary_data.docx]

# **Supplementary Material**

# **Title:** Reducing initial loss to follow up among people with bacteriologically confirmed tuberculosis: LINKEDin, a quasi-experimental study in South Africa.

**Authors**

*Sue-Ann Meehan^1^, Anneke C. Hesseling^1^, Andrew Boulle^2,3^, Jolene Chetty^4^, Lucy Connell^5^, Nomthandazo J. Dlamini-Miti^5^, Rory Dunbar^1^, Karen Du Preez^1^, Gavin George^6,7^, Graeme Hoddinott^1^, Karen Jennings^8^, Florian M. Marx^1,9,10^, Vanessa Mudaly^11^, Pren Naidoo^12^, Neo Ndlovu^5^, Jacqueline Ngozo^13^, Mariette Smith^2,3^, Michael Strauss^6^, Gaurang Tanna^12^, Nosivuyile Vanqa^1^, Arne von Delft^2,3^, Muhammad Osman^1,14^

**Affiliations**

^1^Desmond Tutu TB Centre, Department of Paediatrics and Child Health, Faculty of Medicine and Health Sciences, Stellenbosch University, Cape Town, South Africa

^2^Centre for Infectious Disease Epidemiology and Research, School of Public Health and Family Medicine, Faculty of Health Sciences, University of Cape Town

^3^Health Intelligence Directorate, Western Cape Government, Department of Health and Wellness

^4^Interactive Research and Development South Africa (IRD SA), Block A 1st Floor, 36 Wierda Road, Wierda Valley West, Sandton, Johannesburg

^5^Right to Care South Africa, Helen Joseph Hospital, Johannesburg, South Africa

^6^Health Economics and HIV and AIDS Research Division (HEARD), University of KwaZulu-Natal, Durban 4041, South Africa

^7^Division of Social Medicine and Global Health, Lund University, 22363 Lund, Sweden

^8^City of Cape Town Health Department, South Africa

^9^Division of Tropical Medicine, Center for Infectious Diseases, Heidelberg University Hospital, Heidelberg, Germany

^10^DSI-NRF South African Centre of Excellence in Epidemiological Modelling and Analysis (SACEMA), Stellenbosch University, Stellenbosch, South Africa

^11^Western Cape Government, Department of Health and Wellness

^12^Bill and Melinda gates Foundation, Johannesburg, South Africa

^13^ Kwa-Zulu Natal Department of Health and Wellness

^14^School of Human Sciences, University of Greenwich, London, UK

**Supplementary table 1: Key contextual differences between districts included in the LINKEDin study.**

| **District, Province** | **Setting** | **Estimated Population (2019)^a^** | **Estimated TB case notification (per 100,000)**  **(2015)^b^** | **DS-TB treatment success among registered patients %**  **(2018)^a^** | **%DS-TB patients died among registered patients (2018)^a^** | **Antenatal**  **HIV**  **prevalence**  **(2019)^c^** |
| --- | --- | --- | --- | --- | --- | --- |
| Ugu, KwaZulu-Natal | Rural | <800,000 | 810 | 75.0 | 10.5 | 44.2 |
| City of Johannesburg, Gauteng | Urban | 5.3 million | 326 | 83.2 | 6.1 | 25.5 |
| City of Cape Town, Western Cape | Urban | 4.1 million | 596 | 76.3 | 3.5 | 22.0 |
| SA Overall |  | 59 million | 360^d^ | 79.2 | 7.0 | 30.0 |

a. District health barometer 2019/2020 (1)

b. District-level estimates of TB incidence have not been published since 2015 (2)

c. The 2019 National Antenatal Sentinel HIV Survey (3)

d. The WHO Global TB Report 2022 provides an estimated incidence for South Africa (4)

|  | Oct-Dec 2018 | | Jan 2019-Dec 2020 | | Relative Reduction ILTFU  (95%CI) |
| --- | --- | --- | --- | --- | --- |
|  | Newly diagnosed PWTB | ILTFU  % (95% CI) | Newly diagnosed PWTB | ILTFU  % (95% CI) |  |
| **Western Cape Province** | **2478** | **593**  **23.93% (22.3, 25.6)** | **16301** | **3340**  **20.5% (19.9,21.1)** | **14.4%**  **(7.6, 20.7)** |
| Tygerberg sub-district | 1332 | 361  27.1% (24.7, 29.5) | 8538 | 2166  25.4% (24.4,26.3) | 6.4%  (-3, 14.9) |
| Tygerberg Hospital | 309 | 148  47.9% (42.3, 53.5) | 1929 | 996  51.6% (49.4,53.9) | -7.8%  (-22, 4.8) |
| Surrounding facilities | 1023 | 213  20.82% (18.3, 23.3) | 6609 | 1170  17.7% (16.8,18.6) | 15%  (3.1, 25.4) |
| Khayelitsha sub-district | 1146 | 232  20.24% (17.9, 22.6) | 7763 | 1174  15.1% (14.3,15.9) | 25.3%  (15.2, 34.2 ) |
| Khayelitsha Hospital | 191 | 85  44.5% (37.5, 51.6) | 1630 | 638  39.1% (36.8,41.5) | 12.1%  (-4.2, 25.8) |
| Surrounding facilities | 955 | 147  15.4% (13.1, 17.7) | 6133 | 536  8.7% (8,9.4) | 43.2%  (32.8, 52.1) |

**Supplementary Table 2: Relative reduction in ILTFU between baseline and intervention periods in the Western Cape Province by sub-district and facility type, using all persons diagnosed with TB including clinically and bacteriologically confirmed as reported by the Provincial Health Data Centre.**

CI: Confidence interval

ILTFU: Initial loss to follow up

**Supplementary Table 3: Relative reduction in ILTFU between baseline and intervention periods in both subdistricts per province, with COVID-19 lockdown period (April, May, June 2020) excluded from the intervention phase**

|  | Oct-Dec 2018 | | Jan 2019-March 2020 and July 2020-Dec 2020 | | Relative Reduction ILTFU  (95%CI) |
| --- | --- | --- | --- | --- | --- |
|  | Newly diagnosed PWTB | ILTFU  % (95% CI) | Newly diagnosed PWTB | ILTFU  % (95%CI) |  |
| KwaZulu-Natal (KZN) | 327 | 81  24.8% (20.1, 29.4) | 1825 | 252  13.8% (12.2,15.4) | 44.3%  (30.5, 55.3) |
| Gauteng (GP) | 921 | 292  31.7% (28.7, 34.7) | 4942 | 1630  33% (31.7,34.3) | -4%  (-15.3, 6.1) |
| Western Cape (WCP) | 1323 | 296  22.37% (20.1, 24.6) | 8628 | 1481  17.2% (16.4,18) | 23.3%  (14.3, 31.3) |

CI: Confidence interval

ILTFU: Initial loss to follow up

**Supplementary Table 4: Relative reduction in ILTFU between baseline and intervention periods by sub-districts across provinces, with COVID-19 lockdown period (April, May, June 2020) excluded from the intervention phase.**

|  | | Oct-Dec 2018 | | Jan 2019-March 2020 and July 2020-Dec 2020 | | Relative Reduction ILTFU  (95%CI) |
| --- | --- | --- | --- | --- | --- | --- |
|  |  | Newly diagnosed PWTB | ILTFU  % (95% CI) | Newly diagnosed PWTB | ILTFU  % (95%CI) |  |
| KwaZulu Natal | Umdoni  (Hospital-recording) | 131 | 33  25.2% 17.8,32.6) | 724 | 120  16.6% (13.9,19.3) | 34.2%  (7.8, 53) |
|  | Ray Nkonyeni  (Alert and Response) | 196 | 48  24.5% (18.5,30.5) | 1101 | 132  12% (10.1,13.9) | 51%  (34.4, 63.5) |
| Gauteng | Region D  (Hospital-recording) | 713 | 208  29.2% (25.8,32.5) | 3737 | 1193  31.9% (30.4,33.4) | -9.4%  (-23.8, 3.3) |
|  | Region E  (Alert and Response) | 208 | 84  40.4% (33.7,47.1) | 1205 | 437  36.3% (33.6,39) | 10.2%  (-7.7, 25.1) |
| Western Cape | Tygerberg  (Hospital-recording) | 761 | 185  24.3% (21.3,27.4) | 4696 | 977  20.8% (19.6,22) | 14.4%  (1.8, 25.4) |
|  | Khayelitsha  (Alert and Response) | 562 | 111  19.75% (16.5,23) | 3932 | 504  12.8% (11.8,13.9) | 35.1%  (21.9, 46.1) |

CI: Confidence interval

ILTFU: Initial loss to follow up

|  | | Oct-Dec 2018 | | Jan 2019-March 2020 and July 2020-Dec 2020 | | Relative Reduction ILTFU  (95%CI) |
| --- | --- | --- | --- | --- | --- | --- |
|  |  | Newly diagnosed PWTB | ILTFU  % (95% CI) | Newly diagnosed PWTB | ILTFU  % (95%CI) |  |
| **Sub-districts implementing the hospital-recording intervention (no intervention in surrounding facilities)** | | | | | | |
| Umdoni  (KwaZulu-Natal) | GJ Crookes Hosp | 65 | 23  35.4% (23.8, 47) | 319 | 65  20.4% (16,24.8) | 42.4%  (14.6, 61.2) |
|  | Surrounding PHC facilities | 66 | 10  15.2% (6.5, 23.8) | 405 | 55  13.6% (10.2,16.9) | 10.4%  (-66.9, 51.9) |
| Region D (Gauteng) | CH Baragwanath Hosp | 169 | 94  55.6% (48.1, 63.1) | 1060 | 678  64% (61.1,66.9) | -15%  (-32.6, 0.2) |
|  | Surrounding PHC facilities | 544 | 114  21.0% (17.5, 24.4) | 2677 | 515  19.2% (17.7,20.7) | 8.2%  (-10, 23.4) |
| Tygerberg (Western Cape) | Tygerberg Hospital | 173 | 74  42.8% (35.4, 50.1) | 1040 | 467  44.9% (41.9,47.9) | -5%  (-26.3, 12.8) |
|  | Surrounding PHC facilities | 588 | 111  18.9% (15.7, 22) | 3656 | 510  13.9% (12.8,15.1) | 26.1%  (11, 38.6) |
| **Sub-districts implementing the alert-and-response patient management intervention** | | | | | | |
| Ray Nkonyeni (KwaZulu-Natal) | Gamalakhe CHC | 59 | 7  11.9% (3.6, 20.1) | 374 | 41  11% (7.8,14.1) | 7.6%  (-96.2, 56.5) |
|  | Surrounding PHC facilities | 137 | 41  29.9% (22.3, 37.6) | 727 | 91  12.5% (10.1,14.9) | 58.2%  (42.4, 69.6) |
| Region D (Gauteng) | Edenvale Hospital | 59 | 43  72.9% (61.5, 84.2) | 377 | 240  63.7% (58.8,68.5) | 12.7%  (-3.9, 26.6) |
|  | Surrounding PHC facilities | 149 | 41  27.5% (20.3, 34.7) | 828 | 197  23.8% (20.9,26.7) | 13.5%  (-15.3, 35.2) |
| Khayelitsha (Western Cape) | Khayelitsha Hospital | 79 | 22  27.8% (18.0, 37.7) | 688 | 215  31.3% (27.8,34.7) | -12.2%  (-62.8, 22.6) |
|  | Surrounding PHC facilities | 483 | 89  18.4% (15.0, 21.9) | 3244 | 289  8.9% (7.9,9.9) | 51.7%  (39.9, 61.1) |

**Supplementary Table 5: Relative reduction in ILTFU between baseline and intervention periods by place of diagnosis for sub-districts by intervention type, with COVID-19 lockdown period (April, May, June 2020) excluded from the intervention phase.**

CI: Confidence interval, ILTFU: Initial loss to follow up

**References**

1. Massyn N, Barron P, Day C, Ndlovu N, Padarath A, editors. District Health Barometer 2018/19. Durban, South Africa; 2020.

2. Massyn N, Peer N, English R, Padarath A, Barron P, Day C. District Health Barometer 2015/16. Durban: Health Systems Trust; 2016.

3. Woldesenbet SA, Lombard C, Manda S, Kufa T, Ayalew K, Cheyip M, et al. The 2019 National Antenatal HIV Sentinel Survey. 2021.

4. World Health Organization. Global Tuberculosis Report 2022. Geneva, Switzerland; 2022.
